# Strategies for handling missing data in randomised trials

**DOI:** 10.1186/1745-6215-12-S1-A59

**Published:** 2011-12-13

**Authors:** Ian R White

**Affiliations:** 1 MRC Biostatistics Unit, Cambridge CB2 0SR, UK

## 

Missing outcome data in randomised trials are a major potential source of bias in trial results, and their correct handling can be a major source of difficulty for investigators [1]. Missing outcome data matter for three main reasons:

1. They lead to a loss of power. This cannot be reversed, and so all efforts should be made to maximise completeness of follow-up.

2. Any analysis of incomplete data makes untestable assumptions and is likely to be biased if those assumptions do not hold. Because the correct assumption is usually unknown, it is important to justify the assumption used from subject-matter knowledge, and to perform sensitivity analyses.

3. Some popular analysis methods give inefficient estimates and/or biased standard errors, even if their assumptions are correct.

This talk will outline some of the statistical methods available for handling missing data in randomised trials, and what their underlying assumptions are. Some methods are simple to implement but harder to support: for example, "Last Observation Carried Forward" is based on an assumption that is rarely justified, while analysis of complete cases can be statistically inefficient. More complex methods such as mixed models and multiple imputation are usually based around a missing at random assumption, which is popular because it is perceived to become more plausible as models become more complex.

The intention-to-treat principle is sometimes seen as requiring missing values to be imputed. By focussing instead on assumptions, I will show that it is sufficient for all *observed* data to be included in the main analysis, but that sensitivity analyses must take account of all randomised individuals. This approach will be summarised in an intention-to-treat analysis strategy [2].

I will also briefly describe how to perform a sensitivity analysis and how to handle missing baseline variables.
